# Improved Thermal Sensitivity Using Virtual Monochromatic Imaging Derived from Photon Counting Detector CT Data Sets: Ex Vivo Results of CT-Guided Cryoablation in Porcine Liver

**DOI:** 10.1007/s00270-023-03546-3

**Published:** 2023-09-12

**Authors:** Josua A. Decker, Franka Risch, Florian Schwarz, Christian Scheurig-Muenkler, Thomas J. Kroencke

**Affiliations:** 1 Department of Diagnostic and Interventional Radiology, University Hospital Augsburg, Stenglinstr. 2, 86156 Augsburg, Germany; 2 Medical Faculty, Ludwig Maximilian University Munich, Bavariaring 19, 80336 Munich, Germany; 3 Centre for Advanced Analytics and Predictive Sciences, Augsburg University, Universitätsstr. 2, 86159 Augsburg, Germany; 4 Diagnostic and Interventional Radiology, Donauisar Klinikum Deggendorf, Perlasberger Str. 41, 94469 Deggendorf, Germany

**Keywords:** Cryoablation, Thermal analysis, Liver, Computed tomography

## Abstract

**Purpose:**

To investigate differences in thermal sensitivity of virtual monoenergetic imaging (VMI) series generated from photon-counting detector (PCD) CT data sets, regarding their use to improve discrimination of the ablation zone during percutaneous cryoablation.

**Materials and Methods:**

CT-guided cryoablation was performed using an ex vivo model of porcine liver on a PCD-CT system. The ablation zone was imaged continuously for 8 min by acquiring a CT scan every 5 s. Tissue temperature was measured using fiberoptic temperature probes placed parallel to the cryoprobe. CT-values and noise were measured at the tip of the temperature probes on each scan and on VMI series from 40 to 130 keV. Correlation of CT-values and temperature was assessed using linear regression analyses.

**Results:**

For the whole temperature range of [− 40, + 20] °C, we observed a linear correlation between CT-values and temperature in reference 70 keV images (*R*^2^ = 0.60, *p* < 0.001) with a thermal sensitivity of 1.4^HU^/_°C_. For the most dynamic range of [− 15, + 20] °C, the sensitivity increased to 2.4^HU^/_°C_ (*R*^2^ = 0.50, *p* < 0.001). Using VMI reconstructions, the thermal sensitivity increased from 1.4 ^HU^/_°C_ at 70 keV to 1.5, 1.7 and 2.0^HU^/_°C_ at 60, 50 and 40 keV, respectively (range [− 40, + 20] °C). For [− 15, + 20]°C, the thermal sensitivity increased from 2.4^HU^/_°C_ at 70 keV to 2.5, 2.6 and 2.7^HU^/_°C_ at 60, 50 and 40 keV, respectively. Both CT-values and noise also increased with decreasing VMI keV-levels.

**Conclusion:**

During CT-guided cryoablation of porcine liver, low-keV VMI reconstructions derived from PCD-CT data sets exhibit improved thermal sensitivity being highest between + 20 and − 15 °C.

**Graphical Abstract:**

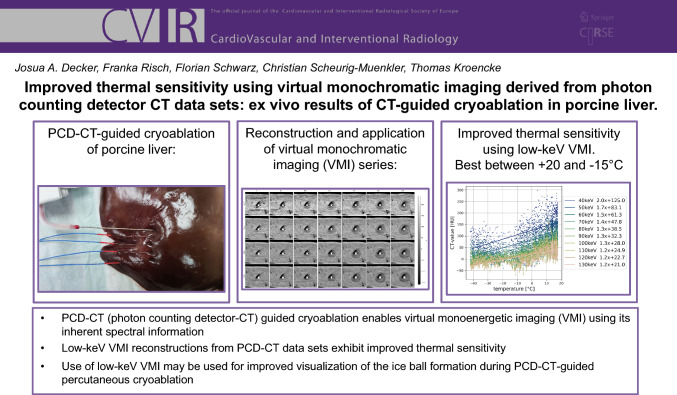

**Supplementary Information:**

The online version contains supplementary material available at 10.1007/s00270-023-03546-3.

## Introduction

Percutaneous cryoablation plays an increasing role in the minimally invasive treatment of liver tumors and hepatic metastases [1–4]. In several studies, computed tomography (CT)-guided percutaneous cryoablation has been reported to be a safe and efficacious treatment option for various liver lesions with low local recurrence rates [5–10]. During CT-guided cryoablation, the forming ice ball is visualized by intraprocedural scanning of the growing hypoattenuating zone (phase transition front, i.e., interface between frozen and unfrozen liver tissue) around the cryoablation probes [11–13]. The temperature at the outer margin of the ice ball is 0° Celsius [14]. The attenuation of the freezing liver tissue decreases with decreasing temperatures and partially shows a linear correlation [11, 15, 16]. Although CT is routinely used in guiding and placing cryoprobes, its use for intraprocedural monitoring and visualization of the ice ball has known limitations [14, 17, 18]. To ensure complete ablation of the target lesion and to prevent injuring adjacent structures, it is important to strive for optimal discrimination of the ice ball and especially its outer margin.

Recently, CT systems with photon-counting detectors (PCD) have been introduced in clinical routine, which generate spectral information for every scan due to their inherent spectral sensitivity [19, 20]. Using this spectral information, virtual monoenergetic image (VMI) reconstructions can be generated, which show an increased soft-tissue contrast at low keV-levels [21, 22]. Low-keV VMI improve the conspicuity of hypoattenuating liver metastases, however it is not yet known, if VMI can also be utilized to improve the visualization of the ice ball formation during percutaneous cryoablation [21].

Therefore, in this study, we investigated if VMI generated from PCD-CT data sets exhibit differences in thermal sensitivity, and whether these VMI reconstructions can be used to improve discrimination of the ice ball during percutaneous cryoablation.

## Materials and Methods

### Experimental Setup

**Fig. 1:**
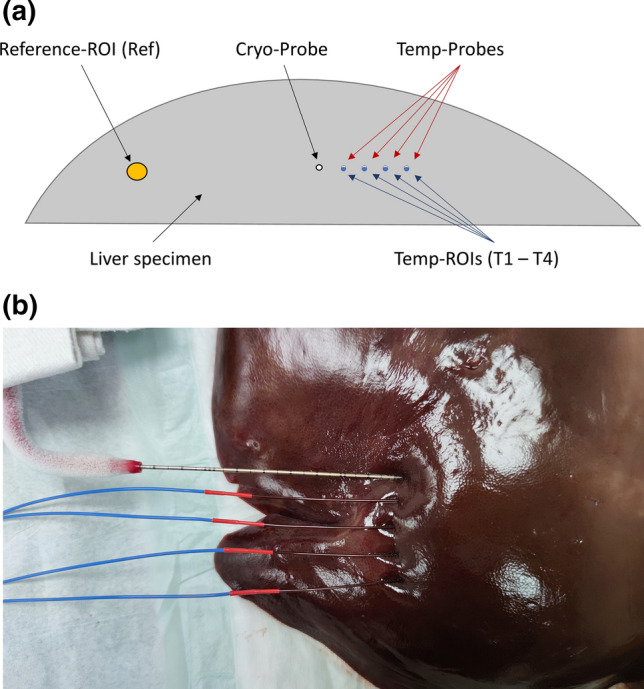
Experimental setup**. a** Schematic of the experimental setup with position of the cryoprobe, the temperature probes, and the locations of the regions of interest (ROIs) for CT-value measurements in equidistance for T1-T4. **b** Picture of the experimental setup with the cryoprobe (upper probe) and the four parallel temperature probes (lower four probes)

A healthy porcine liver was purchased from a local organic butchery and used on the same day. Three experiments were performed in separate liver segments carefully avoiding potential overlapping of puncture and ablation zones. The liver was placed on the CT table surrounded by air. A liquid argon cooled (Linde, Dublin, Ireland) cryoablation system (ICEfx, Icefx; Boston Scientific, Marlborough, Massachusetts, USA) was used for all experiments. The cryoprobe (17 G, IceRod 1.5 CX, Boston Scientific) was placed parallel to the CT table centrally into the liver parenchyma. Four fiber-optic temperature probes (TS3 Sensor, Weidmann Technologies, Germany) were positioned parallel to the cryoprobe in 5 mm intervals with distances of 5, 10, 15 and 20 mm (Fig. 1a and  b). Positioning of the probes was checked by sequential CT acquisitions without table movement. The temperature measurements were obtained using a four-channel thermometry-system (OEM-PLUS RS232, Weidmann Technologies) calibrated with 0.1 °C accuracy from − 100 to + 200 °C.

### Cryoablation Procedure and CT Protocol

The cryoablation procedures were performed on a dual-source PCD-CT scanner (NAEOTOM Alpha, Siemens Healthineers, Erlangen, Germany) with the experimental setup placed in the isocenter. Correct positioning was again verified by a sequential reference CT-scan. After starting continuous temperature measurements at four probe positions (in 0.25 s intervals) and acquisition of a reference CT scan, cryoablation was started with a freezing time of 8 min. During freezing, sequential CT scans without table movement were obtained in 5 s intervals adding up to a total of 97 scans (8*60 s/5 s + 1 at time point 0) for each of the three repetitions.

The following scan parameters were applied: total collimation of 144 × 0.4 mm resulting in 57.6 mm acquisition length in z-axis direction, reference tube potential of 70 kVp, reference tube current time product of 80 mAs, spectral acquisition mode (QuantumPlus, using thresholds at 20, 35, 65 and 70 keV, Siemens Healthineers).

### Image Reconstruction and Image Analysis

The time resolved scans were reconstructed at the scanner console using a smooth body regular kernel (Br40) with an iterative metal artifact reduction (iMAR) at varying VMI levels, ranging from 40 to 130 keV in 10 keV increments. Slice thickness and increment were both 1.0 mm, and the matrix size was 512 pixels with a field of view identical for all three experiments.

For image analysis, regions of interest (ROIs) of 3 mm diameter were placed at the same axial slice position in front of the temperature probe endings (T1-T4) in most homogeneous and least artifact affected tissue using an open-source software (ImageJ version 1.53 k, https://imagej.nih.gov/ij). A reference ROI (ref) with 8 mm diameter was placed at a distance where the tissue was not affected by the cryoablation. Mean and standard deviation of CT-values was automatically derived for all three experiments, the 97 points in time, the 10 VMI levels and all five ROIs at three adjacent slices, respectively, using a python (version 3.9.7) script. For further analysis, ROIs were averaged across the three slices to increase robustness to local CT value inhomogeneities and noise. The signal-to-noise ratio was calculated from the ref ROI measurements, dividing the mean of the CT-values by their standard deviation. For visualization, linear regression results were used to allocate temperatures to CT-values creating ‘temperature-coded’ images.

### Statistical Analysis

Statistical analyses were performed using python (version 3.9.7). The Shapiro–Wilk test was used to test for normal distribution of data. Continuous parametric data are given as mean ± standard deviation, non-parametric data as median with interquartile range. To test for differences, the paired t-test and the Wilcoxon signed rank test were used for parametric and non-parametric data, respectively. Temperature measurements were correlated with the scan points of time and a linear regression performed. All *p*-values were corrected with the Bonferroni method, in case of multiple comparisons. *P*-values < 0.05 were considered to indicate statistical significance.

## Results

### Temperature Measurements

**Fig. 2:**
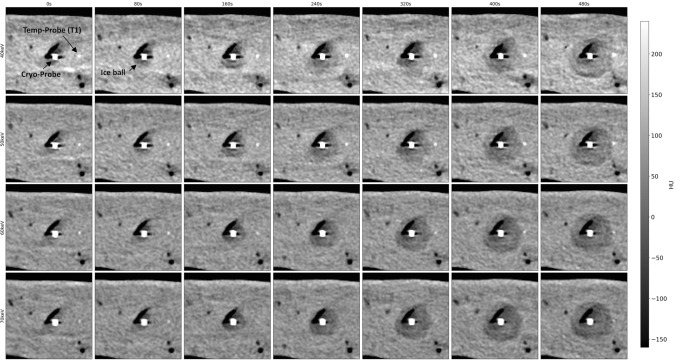
Visualization of the same axial slice during the cryoablation procedure at exemplary time points. On the horizontal axis, time varies from 0 to 480 s in 80 s intervals; on the vertical axis, the level of monoenergetic imaging varies from 40 to 70 keV in 10 keV intervals. HU values of all images are normalized to the grayscale shown on the right

**Fig. 3:**
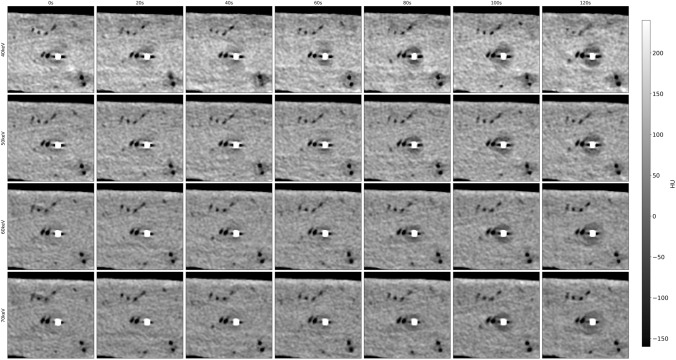
Visualization of the same axial slice during the cryoablation process. On the horizontal axis, time varies from 0 to 120 s in 20 s intervals; on the vertical axis, the level of monoenergetic imaging varies from 40 to 70 keV in 10 keV intervals

Measured temperature shows an exponential decay with the lowest temperatures measured more closely to the tip of the cryoprobe. The lowest measured temperature was − 41.1 °C in about 5 mm distance to the cryoprobe after 8 min of cooling. During ablation, we observed the characteristic low-attenuating area originating from the tip of the cryoprobe. Figure 2 and Fig. 3 illustrate grayscale images for different VMI series over different time scales. Here, the ice ball formation can be observed with better delineation in lower VMI series, which is especially pronounced during the first 120 s of the ablation procedure (see Fig. 3).

### CT-Value Measurements

**Fig. 4:**
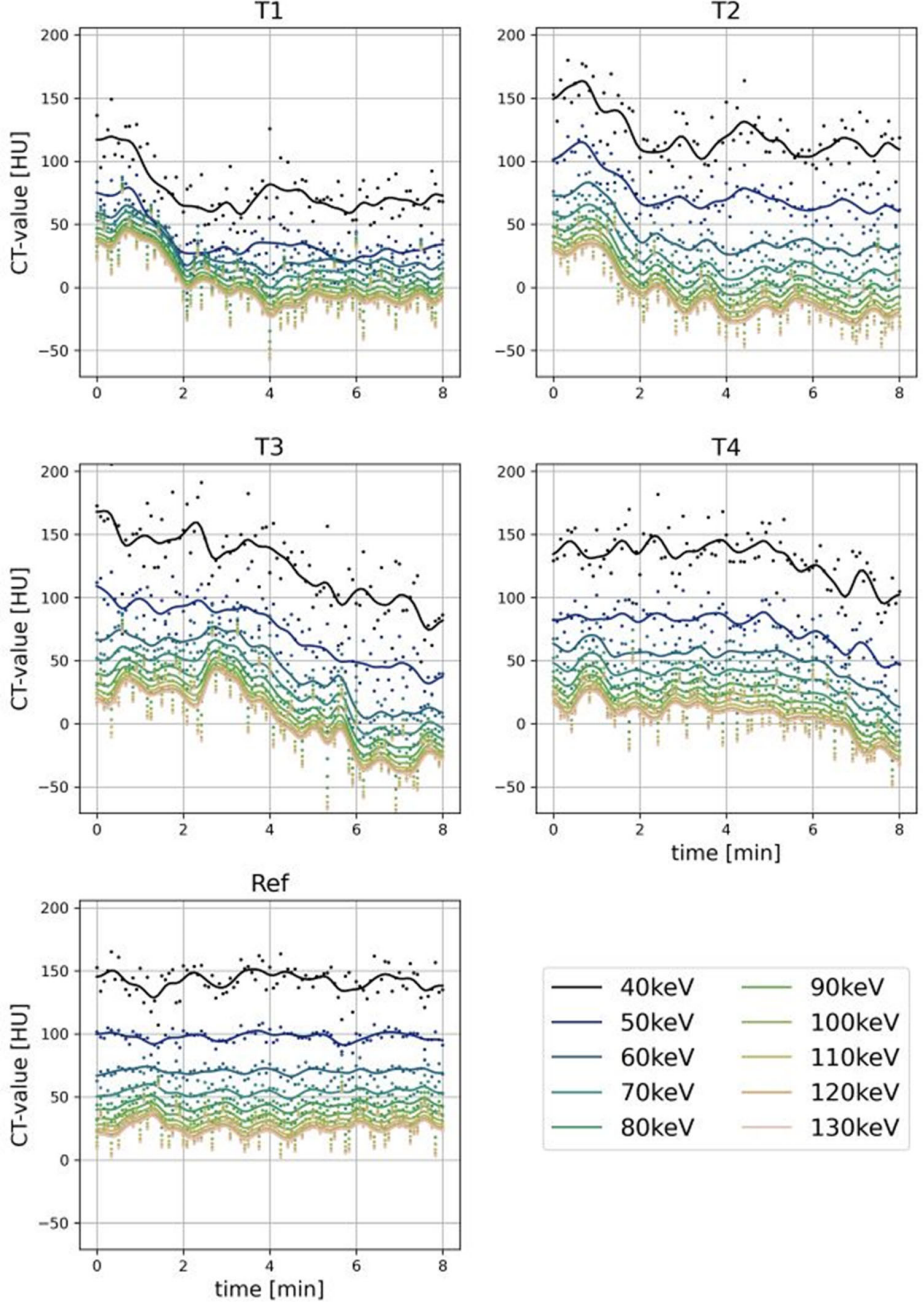
CT-values over time for different regions of interest (ROIs). T1–T4 = ROIs of temperature probes 1–4 with increasing distance to the cryoprobe. Ref = reference ROI

For analysis of the temperature-dependent changes of CT-values, 34,920 ROI measurements were included. Detailed data on CT-values, noise, and SNR for different measurement locations and VMI reconstructions are presented in supplemental Tables 1–3. Figure 4 shows CT-values over time for different distances to the cryoprobe. In the ROIs more closely to the cryoprobe, we observed a more rapid decrease in CT-values compared to the more distant areas, where delayed decrease was observed. In the reference ROI, we observed no relevant changes in attenuation. Therefore, mean CT-values were lower in close proximity to the cryoprobe, which also results in a decreased signal-to-noise ratio at these locations. Regarding virtual monoenergetic levels, we observed an increase in image noise at lower keV-levels exemplarily increasing by 56.8% (SD: 17.6 to 27.6 HU; *p* < 0.001) between 70 and 40 keV series at temperature ROI 1 (T1) in closest proximity to the cryoprobe. CT-values also increased with decreasing keV, exemplarily in the reference ROI with a 117.4% increase (mean: 69.3 to 150.7 HU; *p* < 0.001) between 70 and 40 keV. Because CT-values showed a disproportional increase at lower keV VMI, the overall SNR also increased at lower keV levels. Exemplarily, SNR increased by 80% (1.0 to 1.8, *p* < 0.001) between 70 and 40 keV at T1. Due to the lower CT-values at ROIs more closely to the cryoprobe, overall noise and SNR were also lower in these locations.

### Correlation of Temperature and CT-Values Measurements

**Fig. 5:**
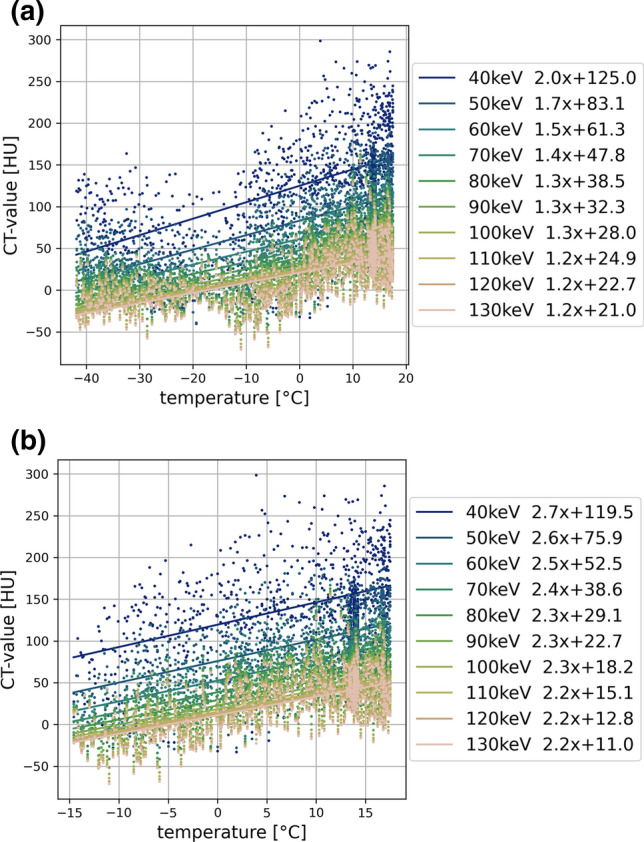
Scatter plots and linear correlation of CT-values and temperatures measured during cryoablation of porcine liver tissue. **a** temperature range of [− 45, + 20] °C. **b** temperature range of [− 15, + 20] °C

**Fig. 6:**
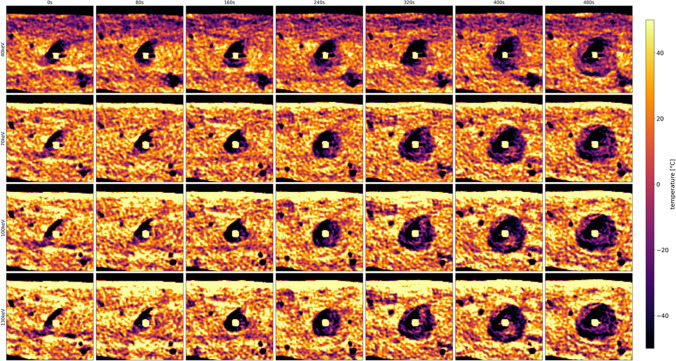
Temperature-coded images, where each voxel in each series was assigned the temperature according to the linear correlation or temperature range of [− 45, + 20] °C (Fig. 5A)

When comparing the measured CT-values with the temperature, we found linear correlations for CT-values and temperature for all VMI. In regression analysis considering the full temperature range [− 40, + 20] °C, this yielded a linear correlation between CT-values and temperature in reference 70 keV images (*R*^2^ = 0.60, *p* < 0.001) with a slope of 1.4, which corresponds to a 1.4 HU decrease per °C. The slope, however, was steeper at lower keV-levels increasing to 1.5, 1.7 and 2.0 ^HU^/_°C_ for 60, 50 and 40 keV, respectively (Fig. 5a). In the scatter plots, we observed only minor changes to a plateau-like configuration of CT-values below − 15 °C. Therefore, we additionally assessed correlation of CT-values and temperature for the dynamic range of [− 15, 20] °C. Applying this range, we also found a linear correlation in reference 70 keV images (*R*^2^ = 0.50, *p* < 0.001) with a steeper slope of 2.4 ^HU^/_°C_ increasing to 2.5, 2.6 and 2.7 ^HU^/_°C_ for 60, 50 and 40 keV, respectively (Fig. 5b). Figure 6 shows temperature-coded images, where each voxel in each series was assigned the temperature according to the calculated linear correlation derived from regression analysis.

## Discussion

In this study, we investigated the thermal sensitivity of VMI series of various keV-levels generated from photon-counting detector CT data sets in an ex vivo porcine liver model during CT-guided cryoablation. The main findings are: (1) There is a linear correlation of tissue CT-values and temperature, especially between + 20 and − 15 °C with formation of a plateau at lower temperatures; (2) VMI reconstructions at lower keV-levels show higher image noise but also higher signal-to-noise ratios; (3) with decreasing keV-levels, we observed a more pronounced linear correlation of CT-values and temperature, which (4) results in an increased difference of CT-values between the ablation zone and the surrounding tissue (especially at earlier time points, when the desired temperature has not yet been reached).

Image guidance in percutaneous cryoablation involves different steps such as planning, targeting, monitoring and assessment of treatment response [23]. Tissue changes that occur during the procedure can be monitored by CT to assess adequate tumor coverage, affection of nearby normal (non-target) structures and may also be used to perform intraprocedural modifications [14]. Careful observation of the hypoattenuating leading edge of the ice ball is required to achieve the best possible treatment and to avoid recurrences [12, 13]. Evaluating the potential of dual-energy CT for cryoablation guidance in bone, Morris et al. reported an earlier ice ball visualization in the spine and pelvis [24]. But how can spectral information be used to improve discrimination of the ablation zone in hepatic tissue? Since recently introduced photon-counting detectors inherently provide spectral information for each scan, it is necessary to investigate, whether and how this additional and directly accessible information can be used to improve the peri-interventional control of the ablation zone in patients undergoing percutaneous cryoablation.

Using an ex vivo porcine liver, we observed a strong linear correlation of temperature and CT-values that was even more distinct on VMI series at low keV-levels. It is not unknown that there is a correlation between temperature and CT attenuation of hepatic tissue. Using a similar experimental setup, Huebner et al. reported a thermal sensitivity of 0.95 ^HU^/_°C_ (*R*^2^ = 0.73) in an ex vivo porcine liver model [15]. Pohlan et al. reported a thermal sensitivity of even 2.11 ^HU^/_°C_ (*R*^2^ = 0.55) using porcine liver placed on porcine ribs [11]. At comparable keV-levels, our experiments yielded a thermal sensitivity of 1.2 ^HU^/_°C_ (*R*^2^ = 0.46) for the temperature range of [− 40, 20] °C. An explanation for the observed deviations might be that Huebner et al. used temperatures as low as − 75.4 °C compared to − 41.1 °C in our study and about − 35 °C in Pohlan et al. Due to the plateau below − 20 °C, which can also be observed in the study of Huebner et al., the influence of even lower temperatures will most likely attribute to a flattening of the correlation line translating into a reduction thermal sensitivity. Additionally, we used a reference tube voltage of 70 kVp compared to 120 kVp of Huebner et al. and Pohlan et al. [11, 15]. Due to the stagnating CT-values at temperatures below − 20 °C, we decided to separately assess the thermal sensitivity of this range of transition, where we calculated a significantly higher value of 2.2 ^HU^/_°C_ (*R*^2^ = 0.40) at 120 keV VMI. Utilizing the inherent spectral information of the photon-counting detector data sets, we showed that thermal sensitivity could be further improved (up to 2.7 ^HU^/_°C_) by lowering keV-levels to 40 keV.

But how can these observations be utilized in clinical routine? First, it must be noted that low-keV VMI reconstructions can directly be generated and displayed on the scanner during ablation. These low-keV VMI reconstructions (with shown increased thermal sensitivity) could then be used during PCD-CT-guided cryoablation for a more precise delineation of the ice ball and subsequent ablation zone. This could help facilitating complete coverage of the target lesion and avoiding damage to adjacent structures. Furthermore, the highest thermometric sensitivity between 20 and − 15 °C in combination with VMI reconstructions may be used for intraprocedural modification of the ablation zone by means of duty cycle setting adjustments at earlier time points – maybe also with the help of temperature-coded images. In consequence, the cryoablation of tumors could possibly be performed with greater confidence without additional effort, thanks to the routinely available VMI reconstructions derived from the inherent spectral PCD-data.

This study has several limitations. First, we investigated an ex vivo porcine liver model without perfusion. Second, the experimental setup was surrounded by air, and third, we performed the ablation on non-malignant tissue. Therefore, the results cannot simply be translated to an in vivo intervention. Fourth, we did not investigate the thermal sensitivity for increasing temperatures. Other studies also investigated the thermal sensitivity of CT for higher temperatures with the use of radiofrequency, microwave and laser ablation [11, 25–29]. However, since heating is accompanied with fundamentally different changes of the affected tissue (such as gas building and irreversible protein denaturation and charring), we did not compare our findings to studies investigating CT thermal sensitivity by heating hepatic tissue [29]. Last, we did not investigate, whether these findings translate into an earlier visibility of the ablation zone in a real ablation scenario. This impact should be evaluated in future studies before VMI reconstructions may be routinely used during hepatic cryoablation.

In conclusion, this experimental study provides evidence that routinely and directly available low-keV VMI reconstructions from inherently spectral PCD-CT data sets can be used to improve the thermal sensitivity of CT during cryoablation of liver tissue. Additional studies are necessary to assess, how these findings can be translated into an in vivo ablation and clinical routine.

### Supplementary Information

Below is the link to the electronic supplementary material.

Supplementary file1 (DOCX 21 KB)

## Abbreviations

CT: Computed tomography
PCD: Photon-counting detector
ROI: Region of interest
T1–T4: Temperature probes number 1–4
VMI: Virtual monoenergetic images
