# Supplementary material for: Improved Thermal Sensitivity Using Virtual Monochromatic Imaging Derived from Photon Counting Detector CT Data Sets: Ex Vivo Results of CT-Guided Cryoablation in Porcine Liver
Source: Cardiovasc Intervent Radiol. 2023 Sep 12;46(10):1385–93. doi: 10.1007/s00270-023-03546-3 (PMC10547619; doi:10.1007/s00270-023-03546-3)
Supplement: Supplementary file 1 — Supplementary file1 (DOCX 21 KB) [file 270_2023_3546_MOESM1_ESM.docx]

**Supplemental Tables**

| **Supplemental Table 1** CT-values (signal; Hounsfield units) for different regions of interest and different virtual monoenergetic imaging levels. | | | | | |
| --- | --- | --- | --- | --- | --- |
| **keV** | **T1** | **T2** | **T3** | **T4** | **Reference** |
| 40 | 49.1 ± 36.4 | 143.4 ± 27.4 | 153.9 ± 45.8 | 157.4 ± 43.2 | 150.7 ± 20.5 |
| 50 | 25.5 ± 28.4 | 93.0 ± 21.9 | 104.1 ± 35.6 | 109.2 ± 36.3 | 112.5 ± 7.2 |
| 60 | 21.4 ± 25.5 | 64.7 ± 24.7 | 74.1 ± 31.6 | 84.0 ± 31.9 | 85.5 ± 5.7 |
| 70 | 17.6 ± 25.8 | 47.9 ± 26.5 | 56.7 ± 29.5 | 68.0 ± 30.3 | 69.3 ± 9.8 |
| 80 | 13.8 ± 26.5 | 37.1 ± 27.8 | 45.1 ± 29.4 | 57.3 ± 30.1 | 58.7 ± 12.9 |
| 90 | 11.2 ± 27.2 | 29.9 ± 28.8 | 37.4 ± 29.8 | 50.0 ± 30.2 | 54.3 ± 15.0 |
| 100 | 9.3 ± 27.7 | 25.0 ± 29.5 | 32.1 ± 30.2 | 45.0 ± 30.4 | 51.3 ± 16.5 |
| 110 | 7.9 ± 28.2 | 21.5 ± 30.0 | 28.3 ± 30.5 | 41.4 ± 30.6 | 49.1 ± 17.6 |
| 120 | 6.9 ± 28.5 | 19.1 ± 30.3 | 25.5 ± 30.9 | 38.8 ± 30.8 | 47.5 ± 18.4 |
| 130 | 6.1 ± 28.7 | 17.2 ± 30.6 | 23.4 ± 31.1 | 36.8 ± 30.9 | 46.3 ± 19.0 |
| Data are averaged over all acquisitions and presented as mean ± standard deviation. T1 – T4 = temperature probes 1 – 4. | | | | | |

| **Supplemental Table 2** Noise (standard deviation of CT-values; Hounsfield units) for different regions of interest and different virtual monoenergetic imaging levels. | | | | | |
| --- | --- | --- | --- | --- | --- |
| **keV** | **T1** | **T2** | **T3** | **T4** | **Reference** |
| 40 | 27.6 ± 6.6 | 35.1 ± 14.2 | 31.6 ± 12.0 | 30.3 ± 10.4 | 37.3 ± 4.9 |
| 50 | 23.4 ± 5.1 | 33.6 ± 11.2 | 27.7 ± 8.5 | 27.1 ± 7.6 | 32.9 ± 3.3 |
| 60 | 18.9 ± 4.6 | 26.1 ± 7.4 | 22.5 ± 6.1 | 23.1 ± 7.2 | 28.3 ± 3.2 |
| 70 | 17.6 ± 4.4 | 23.2 ± 6.7 | 21.4 ± 5.8 | 22.1 ± 7.3 | 27.0 ± 3.3 |
| 80 | 17.3 ± 4.4 | 22.6 ± 6.7 | 20.9 ± 5.9 | 21.7 ± 7.7 | 26.5 ± 3.5 |
| 90 | 17.2 ± 4.5 | 22.6 ± 6.9 | 20.7 ± 6.1 | 21.5 ± 8.0 | 26.3 ± 3.6 |
| 100 | 17.2 ± 4.6 | 22.7 ± 7.1 | 20.6 ± 6.3 | 21.4 ± 8.3 | 26.2 ± 3.8 |
| 110 | 17.2 ± 4.6 | 23.0 ± 7.4 | 20.6 ± 6.4 | 21.4 ± 8.5 | 26.1 ± 3.9 |
| 120 | 17.2 ± 4.7 | 23.0 ± 7.5 | 20.6 ± 6.6 | 21.4 ± 8.6 | 26.1 ± 4.0 |
| 130 | 17.3 ± 4.8 | 23.2 ± 7.7 | 20.6 ± 6.7 | 21.4 ± 8.7 | 26.1 ± 4.0 |
| Data are averaged over all acquisitions and presented as mean ± standard deviation. T1 – T4 = temperature probes 1 – 4. | | | | | |

| **Supplemental Table 3** Signal-to-noise ratio for different regions of interest and different virtual monoenergetic imaging levels. | | | | | |
| --- | --- | --- | --- | --- | --- |
| **keV** | **T1** | **T2** | **T3** | **T4** | **Reference** |
| 40 | 1.8 ± 1.5 | 3.9 ± 2.0 | 4.8 ± 1.7 | 5.1 ± 1.7 | 4.7 ± 0.7 |
| 50 | 1.1 ± 1.4 | 2.7 ± 1.6 | 4.0 ± 1.5 | 4.0 ± 1.4 | 3.8 ± 0.6 |
| 60 | 1.2 ± 1.6 | 2.4 ± 1.5 | 3.5 ± 1.6 | 3.6 ± 1.4 | 3.3 ± 0.6 |
| 70 | 1.0 ± 1.7 | 2.0 ± 1.5 | 2.8 ± 1.6 | 3.3 ± 1.5 | 2.7 ± 0.5 |
| 80 | 0.8 ± 1.7 | 1.8 ± 1.6 | 2.3 ± 1.6 | 2.8 ± 1.5 | 2.3 ± 0.6 |
| 90 | 0.7 ± 1.7 | 1.5 ± 1.6 | 2.0 ± 1.6 | 2.5 ± 1.5 | 2.1 ± 0.7 |
| 100 | 0.5 ± 1.8 | 1.3 ± 1.7 | 1.7 ± 1.7 | 2.2 ± 1.5 | 2.0 ± 0.8 |
| 110 | 0.5 ± 1.8 | 1.2 ± 1.7 | 1.5 ± 1.7 | 2.1 ± 1.5 | 1.9 ± 0.8 |
| 120 | 0.4 ± 1.8 | 1.1 ± 1.7 | 1.4 ± 1.7 | 2.0 ± 1.5 | 1.9 ± 0.8 |
| 130 | 0.3 ± 1.8 | 1.0 ± 1.7 | 1.3 ± 1.7 | 1.9 ± 1.5 | 1.8 ± 0.8 |
| Data are averaged over all acquisitions and presented as mean ± standard deviation. T1 – T4 = temperature probes 1 – 4. | | | | | |

| **Supplemental Table 4** Results of linear regression analyses of CT-values and temperature [-40, +20]°C for different virtual monoenergetic imaging levels. | | | | | | |
| --- | --- | --- | --- | --- | --- | --- |
| **keV** | **slope** | **y-intercept** | **R²** | **R** | **95% CI** | **p-value** |
| 40 | 2.0 | 125.0 | 0.35 | 0.61 | [0.58 0.65] | <0.0001 |
| 50 | 1.7 | 83.1 | 0.46 | 0.69 | [0.65 0.72] | <0.0001 |
| 60 | 1.5 | 61.3 | 0.54 | 0.73 | [0.70 0.76] | <0.0001 |
| 70 | 1.4 | 47.8 | 0.56 | 0.74 | [0.71 0.76] | <0.0001 |
| 80 | 1.3 | 38.5 | 0.54 | 0.73 | [0.70 0.75] | <0.0001 |
| 90 | 1.3 | 32.3 | 0.52 | 0.71 | [0.68 0.74] | <0.0001 |
| 100 | 1.3 | 28.0 | 0.50 | 0.70 | [0.67 0.73] | <0.0001 |
| 110 | 1.2 | 24.9 | 0.48 | 0.69 | [0.66 0.72] | <0.0001 |
| 120 | 1.2 | 22.7 | 0.46 | 0.68 | [0.65 0.71] | <0.0001 |
| 130 | 1.2 | 21.0 | 0.45 | 0.67 | [0.64 0.70] | <0.0001 |
| 95% CI = 95% confidence interval. | | | | | | |

| **Supplemental Table 5** Results of linear regression analyses of CT-values and temperature [-15, +20°C] for different virtual monoenergetic imaging levels. | | | | | | |
| --- | --- | --- | --- | --- | --- | --- |
| **keV** | **slope** | **y-intercept** | **R²** | **R** | **95% CI** | **p-value** |
| 40 | 2.7 | 119.5 | 0.19 | 0.39 | [0.33 0.44] | <0.0001 |
| 50 | 2.6 | 75.9 | 0.31 | 0.47 | [0.42 0.52] | <0.0001 |
| 60 | 2.5 | 52.5 | 0.43 | 0.54 | [0.49 0.58] | <0.0001 |
| 70 | 2.4 | 38.6 | 0.47 | 0.55 | [0.50 0.59] | <0.0001 |
| 80 | 2.3 | 29.1 | 0.46 | 0.53 | [0.49 0.58] | <0.0001 |
| 90 | 2.3 | 22.7 | 0.45 | 0.52 | [0.47 0.57] | <0.0001 |
| 100 | 2.3 | 18.2 | 0.43 | 0.51 | [0.46 0.56] | <0.0001 |
| 110 | 2.2 | 15.1 | 0.42 | 0.50 | [0.45 0.55] | <0.0001 |
| 120 | 2.2 | 12.8 | 0.40 | 0.49 | [0.44 0.54] | <0.0001 |
| 130 | 2.2 | 11.0 | 0.40 | 0.49 | [0.43 0.53] | <0.0001 |
| 95% CI = 95% confidence interval. | | | | | | |
